# Enhanced Application of Transthyretin in Gastric Cancer Management

**DOI:** 10.31662/jmaj.2025-0225

**Published:** 2025-06-20

**Authors:** Masayuki Urabe

**Affiliations:** 1 Gastrointestinal Surgery Division, Department of Surgery, Japanese Red Cross Omori Hospital, Tokyo, Japan

**Keywords:** elderly patients, gastric cancer, lean body mass, sarcopenia, transthyretin

Gastric cancer (GC) remains one of the leading causes of cancer-related mortality worldwide, with prognosis largely dependent on the patient’s nutritional status as well as the tumor stage. Numerous research groups have extensively investigated the clinical relevance of physiological and hematological nutritional parameters in patients undergoing treatment for GC. The study by Morikawa et al. ^(1)^ aligns with these bodies of work, suggesting that prealbumin concentrations, along with the controlling nutritional status score, may serve as useful predictors of postoperative morbidity following GC surgery ^(1)^. In spite of the relatively limited sample size, their results provided valuable reference and would merit further exploration in this area.

Prealbumin, now more commonly referred to as transthyretin (TTR), is a thyroid hormone transport protein synthesized in the liver, initially found in human cerebral fluid in 1942 and in human serum in 1956 ^(2)^. The potential of TTR analyte as a nutritional biomarker was first proposed by Ingenbleek ^(2)^, who subsequently dedicated his research career to exploring various aspects of TTR behaviors. The seminal study conducted by Ingenbleek et al. ^(3)^, published in *The Lancet* in 1972, demonstrated the utility of measuring TTR levels to assess protein-calorie malnutrition―an insight that emerged unexpectedly during the evaluation of thyroid function in malnourished children. This finding spurred further investigations that reinforced the clinical value of TTR as a key biomarker for monitoring nutritional health.

Malnutrition is prevalent among patients with GC, partly due to impaired food intake, tumor-associated inflammation, and catabolism. TTR, as noted by Morikawa et al. ^(1)^, possesses characteristics that make it advantageous for the quantitative evaluation of these conditions. Firstly, TTR responds rapidly to both nutritional improvement and deterioration, making it well-suited for the prompt detection of nutritional risk. Compared to the conventionally used albumin, which has a biological half-life of approximately 20 days, TTR has a significantly shorter half-life of 1-2 days, allowing it to more sensitively reflect changes in nutritional status. TTR can therefore be utilized not only for pretreatment nutritional assessment but also as a reliable indicator of the efficacy of perioperative nutritional and exercise interventions, which are commonly implemented in GC patients to improve the clinical outcomes ^(4)^.

Moreover, TTR serves as a robust biomarker that faithfully reflects lean body mass (LBM) resources. Plasma TTR levels consistently correspond to metabolic fluctuations in LBM across various health and disease states, irrespective of age, sex or ethnicity ^(2)^. Uniquely, TTR outlines the same age- and sex-evolutionary patterns as those of the residual LBM capacity. These patterns are not consistently replicated by other liver-secreted proteins, such as albumin or retinol-binding protein ^(2)^. In the context of GC treatment, clinical outcomes are strongly influenced by reductions in LBM stores, which may be most accurately identified by declines in plasma TTR levels, as they mirror the dynamic alterations in the LBM compartments. Notably, TTR helps detect nutritional risk even among obese individuals with hidden muscle loss and excessive adiposity, a condition known as sarcopenic obesity.

These clinical features of TTR are especially relevant in super-aged societies such as Japan. Remarkably, in Japan, individuals aged 80 years and older account for the highest proportion of GC deaths, representing approximately half of the total GC-related mortality ^(5)^. This striking statistic highlights the importance of non-invasive TTR measurement as a tool for assessing and monitoring sarcopenia trajectories in elderly populations. A low TTR level can serve as an early warning signal for poor clinical outcomes, even in the absence of overt symptoms or other abnormal laboratory findings. Elderly patients with GC and reduced TTR levels may benefit from personalized treatment planning, e.g., specialized nutritional support and less invasive therapeutic approaches. Measuring TTR is a simple and cost-effective surrogate method for grading sarcopenia and guiding a more adapted protocol in routine clinical practice. It fulfills the essential criteria of an informative biomarker and should be more widely incorporated into screening programs for GC management.

## Article Information

### Conflicts of Interest

None
